# Utility of eosinophil peroxidase as a biomarker of eosinophilic inflammation in asthma

**DOI:** 10.1016/j.jaci.2024.03.023

**Published:** 2024-04-23

**Authors:** Monica Tang, Annabelle R. Charbit, Mats W. Johansson, Nizar N. Jarjour, Loren C. Denlinger, Wilfred W. Raymond, Michael C. Peters, Eleanor M. Dunican, Mario Castro, Kaharu Sumino, Serpil C. Erzurum, Suzy A. Comhair, Wendy C. Moore, Bruce D. Levy, Elliot Israel, Wanda Phipatanakul, Brenda R. Phillips, David T. Mauger, Eugene R. Bleecker, Sally E. Wenzel, Merritt L. Fajt, Prescott G. Woodruff, Annette T. Hastie, John V. Fahy

**Affiliations:** a University of California San Francisco, San Francisco; b University of Wisconsin-Madison, Madison; c University College Dublin, Dublin; d University of Kansas, Kansas City; e Washington University in St Louis, St Louis; f Cleveland Clinic, Cleveland; g Wake Forest University, Winston-Salem; h Brigham and Women’s Hospital; i Boston Children’s Hospital, Boston; j Pennsylvania State University College of Medicine, Hershey; k University of Arizona, Tucson, Ariz; l University of Pittsburgh, Pittsburgh

**Keywords:** Asthma, eosinophil, eosinophilic inflammation, sputum, mepolizumab, mucus plugs

## Abstract

**Background:**

The relative utility of eosinophil peroxidase (EPX) and blood and sputum eosinophil counts as disease biomarkers in asthma is uncertain.

**Objective:**

We sought to determine the utility of EPX as a biomarker of systemic and airway eosinophilic inflammation in asthma.

**Methods:**

EPX protein was measured by immunoassay in serum and sputum in 110 healthy controls to establish a normal reference range and in repeated samples of serum and sputum collected during 3 years of observation in 480 participants in the Severe Asthma Research Program 3.

**Results:**

Over 3 years, EPX levels in patients with asthma were higher than normal in 27% to 31% of serum samples and 36% to 53% of sputum samples. Eosinophils and EPX correlated better in blood than in sputum (*r*_s_ values of 0.74 and 0.43, respectively), and high sputum EPX levels occurred in 27% of participants with blood eosinophil counts less than 150 cells/μL and 42% of participants with blood eosinophil counts between 150 and 299 cells/μL. Patients with persistently high sputum EPX values for 3 years were characterized by severe airflow obstruction, frequent exacerbations, and high mucus plug scores. In 59 patients with asthma who started mepolizumab during observation, serum EPX levels normalized in 96% but sputum EPX normalized in only 49%. Lung function remained abnormal even when sputum EPX normalized.

**Conclusions:**

Serum EPX is a valid protein biomarker of systemic eosinophilic inflammation in asthma, and sputum EPX levels are a more sensitive biomarker of airway eosinophilic inflammation than sputum eosinophil counts. Eosinophil measures in blood frequently miss airway eosinophilic inflammation, and mepolizumab frequently fails to normalize airway eosinophilic inflammation even though it invariably normalizes systemic eosinophilic inflammation.

## Capsule summary

Eosinophil peroxidase is a valid biomarker of eosinophilic inflammation in asthma, and its measurement reveals how blood measures of eosinophilic inflammation can miss airway eosinophilic inflammation and how mepolizumab does not always normalize airway eosinophilic inflammation.

## Graphical Abstract

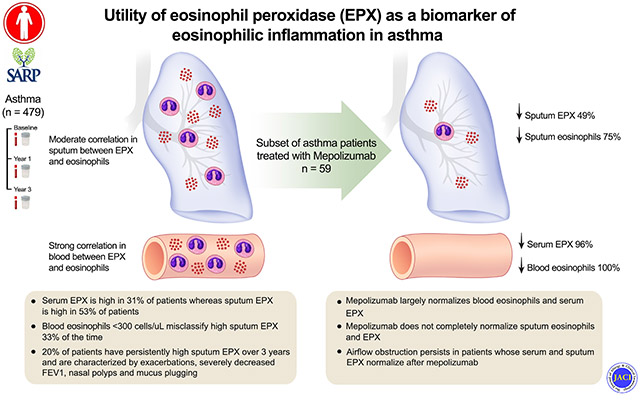

Eosinophils are a key cellular mediator of type 2 immune responses in asthma,^1^ and eosinophil counts in blood are used as a disease classification biomarker to guide decisions about treatments that target the type 2 pathway.^2,3^ Analysis of sputum eosinophil counts is usually restricted to research, although some authors have proposed sputum eosinophil counts as a biomarker to guide asthma treatment.^4,5^ Blood and sputum eosinophil counts are useful biomarkers, but they are imperfect measures of eosinophilic inflammation in the airway.^6,7^ For example, eosinophil counts cannot be measured in stored blood or sputum bio-specimens, and cell counts do not reflect the activation state of eosinophils.^8^ Activated eosinophils release large secondary granules that store eosinophil peroxidase (EPX), major basic protein, eosinophil cationic protein, and eosinophil-derived neurotoxin (EDN).^9^ EPX is the most abundant eosinophil granule protein by weight and is particularly attractive as a protein biomarker of eosinophil activation.^10-14^

EPX has been evaluated previously as a protein biomarker of eosinophilic inflammation in the blood (systemic) and airway compartments in asthma,^11,15-24^ but only 2 studies with limited sample numbers have compared serum and sputum EPX and these studies did not include clinical correlations.^11,21^ In addition, there are no published reports of repeated measures of EPX over time in asthma. We set out here to compare measures of EPX and eosinophil counts in blood and sputum in asthma as biomarkers of systemic and airway eosinophilic inflammation, respectively. We also set out to evaluate how these blood and sputum measures of EPX and eosinophils behave over time in patients with asthma. We leveraged the availability of banked serum and sputum bio-specimens from the Severe Asthma Research Program 3 (SARP-3), a multiyear longitudinal cohort study in patients with asthma, enriched in those with severe disease and designed to advance understanding of molecular mechanisms and molecular phenotypes.^25^ To facilitate our analyses in asthma, we included a healthy cohort to establish a normal reference range for serum and sputum EPX. Furthermore, because a significantly sized subset of SARP-3 patients was started on mepolizumab treatment during their study observation period, we aimed to determine the effects of mepolizumab on eosinophils and EPX levels in blood and sputum.

## METHODS

### Participants

SARP-3 is a multicenter longitudinal cohort study in which 60% of subjects have severe asthma,^25^ as defined according to the European Respiratory Society/American Thoracic Society consensus definition.^4^ Adult patients with asthma were recruited to SARP-3 between November 1, 2012, and April 30, 2015, by 7 clinical research centers (including the University of California San Francisco [UCSF]) in the United States. Data reported here are from 479 participants who provided samples of induced sputum and serum. Aliquots of induced sputum and serum were available from the baseline characterization visit, the 1-year follow-up visit, and the 3-year follow-up visit (see Fig E1 in this article’s Online Repository at www.jacionline.org). For analysis of sputum, we made EPX measures in all available sputum samples over 3 years. For analysis of serum (where sample aliquots were more limited in their availability), we prioritized serum EPX measures from the baseline characterization visit and from (1) visits where participants had companion longitudinal sputum EPX data and (2) visits from participants treated with mepolizumab. Because the SARP-3 protocol included only blood differential cell count analysis at baseline and year 3 visits, these data are reported from only those 2 visits in participants who had a corresponding serum EPX. Additional analyses were performed in a subset of participants with sputum EPX measured at all time points that were not on an eosinophil-targeted biologic and in participants with EPX measures treated with mepolizumab during their enrollment in SARP-3 (see Fig E2 in this article’s Online Repository at www.jacionline.org).

### UCSF healthy subjects.

Seventy-five healthy control subjects had been recruited to research studies between 2005 and 2014. They had no history of pulmonary disease, atopic disease, or allergic rhinitis, and they had normal methacholine responses.

### SARP-3 healthy subjects.

Thirty-five healthy control subjects had no history of pulmonary disease, atopic disease, or allergic rhinitis, and they had normal methacholine responses.

### Clinical characterization

Study participants underwent detailed clinical characterization, including questionnaires, spirometry, fractional exhaled nitric oxide, and optional computed tomography scan of the lungs at regular intervals.^25^ Exacerbations were reported by participants as courses of oral steroids taken for asthma exacerbations. Spirometry was conducted via the SARP network standardized protocol including training, certification, and over-reading. Mucus score was measured as the sum of bronchopulmonary segments with mucus plugs on computed tomography scans of the lungs inspected visually by thoracic radiologists.^26^

### Induced sputum

Sputum induction was conducted using an ultrasonic nebulizer with 3% saline. Sputum was kept on ice or refrigerated and processed within 1 to 4 hours from collection using methods previously described.^7,27^ Briefly, gentle homogenization of whole sputum expectorate was achieved by mixing sputum with 10% di-thiotreitol (Sputolysin, EMD Millipore, Temecula, Calif) in a shaking water bath at 150 shakes/min for 15 minutes. An aliquot of the liquefied sputum was used for cytocentrifugation, and the remainder of the sample was centrifuged at low speed (2000 rpm) for 10 minutes to generate a sputum fluid phase (supernatant). Each center shipped 2 aliquots of sputum supernatant to the UCSF SARP-3 center for measurement of analytes.

### Eosinophil counts in blood and sputum

#### Blood eosinophils.

Differential cell counts in blood, including eosinophil counts, had been determined by the clinical laboratory services at each of the participating SARP-3 sites. Blood eosinophil counts of more than 300 cells/μL were categorized as abnormally high, although we also analyzed blood eosinophils in 4 subgroups (<150 cells/μL, 150-299 cells/μL, 300-449 cells/μL, and >450 cells/μL).^28^

#### Sputum eosinophils.

Cytocentrifuged sputum cells had been stained with Diff-Quik (Siemens Healthcare Ltd, Camber-ley, United Kingdom) and shipped from each SARP-3 center to the sputum cytology core (Wake Forest University, Winston-Salem, NC), where the sputum cells had been counted by light microscopy. A sputum eosinophil percentage of 2 or more was categorized as abnormally high.

### Eosinophil peroxidase

For the study reported here, EPX was measured in serum samples and sputum supernatant samples using a commercially available ELISA (Diagnostics Development, Uppsala, Sweden). The sensitivity is less than 0.1 μg/L, with an assay range from 0 to 25 μg/L. All samples were diluted 1:50 in assay diluent as per manufacturer’s recommendations. We confirmed that the EPX assay did not detect myeloperoxidase (see Fig E3 in this article’s Online Repository at www.jacionline.org). We note that processing may increase EPX levels. A previous study has shown that clotting leads to higher EPX levels in serum than in plasma, but that serum and plasma levels of EPX are strongly correlated.^29^

### Statistical methods

Analyses were performed using R (R Foundation for Statistical Computing, Vienna, Austria) and GraphPad Prism (GraphPad Software, Boston, Mass). *P* values less than .05 were taken as statistically significant. Correlation was calculated using the Spearman correlation. The predictive performance of eosinophil and EPX measures for clinical outcomes was assessed using the area under the receiver-operating characteristic curve. Comparisons between longitudinal subgroups (Sputum EPX-persistently high, Sputum EPX-intermittent, and Sputum EPX-persistently low) were evaluated by chi-square analysis for categorical variables, ANOVA for continuous variables with symmetric distributions, and the Kruskal-Wallis test for continuous variables with skewed distributions. In the longitudinal analysis, comparisons between sputum EPX and FEV_1_ were done using a linear mixed-effects model with a random effect for participant. Comparisons of paired pre- and postmepolizumab data were determined using the McNemar test. Not all participants had data for every study outcome, and analyses used available data.

## RESULTS

### EPX levels are higher in sputum than in blood

**FIG 1.**
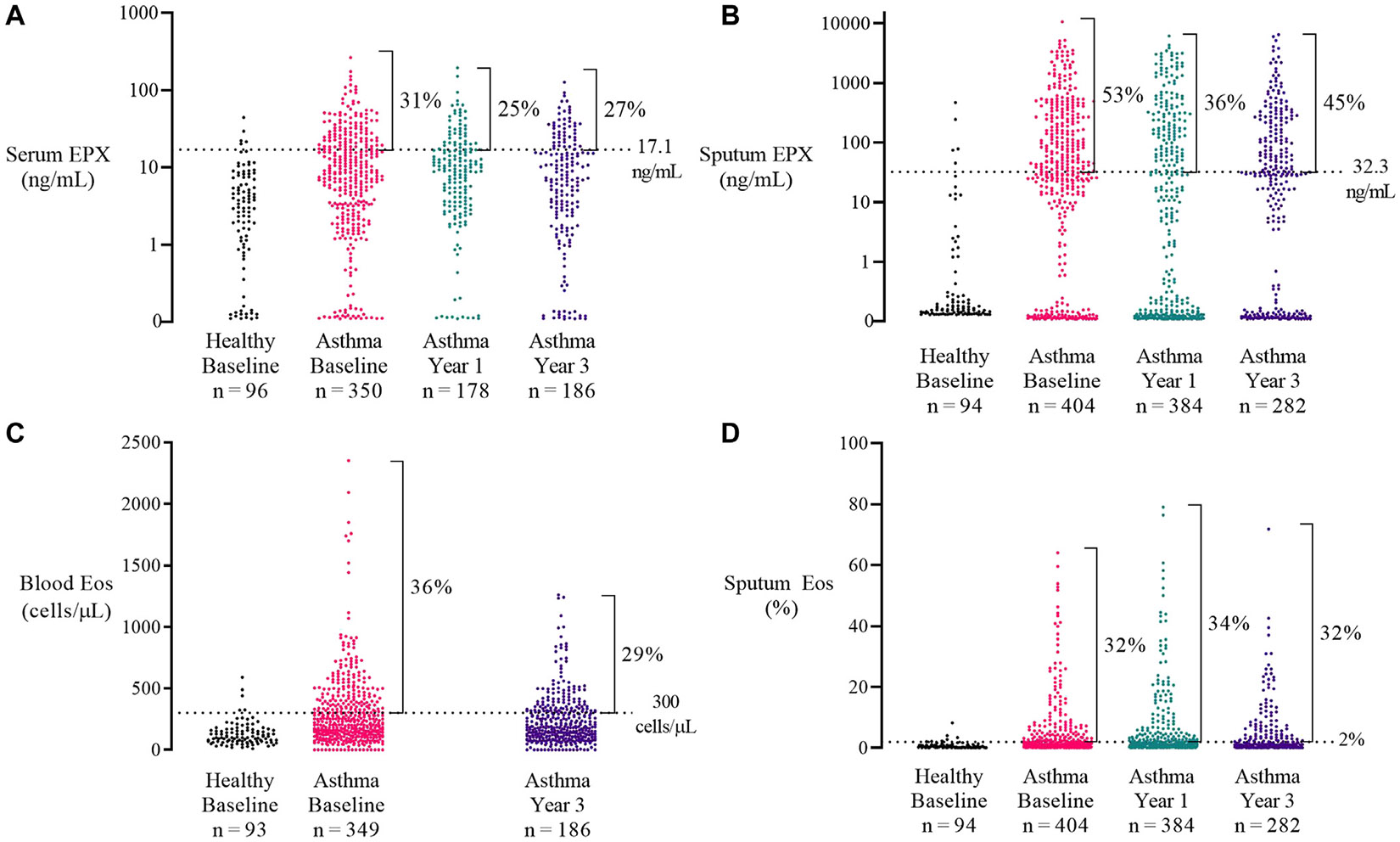
EPX levels are higher in sputum than in serum in patients with asthma. **A,** Serum EPX in healthy controls at baseline and patients with asthma at baseline, year 1, and year 3. **B,** Sputum EPX in healthy controls at baseline and participants with asthma at baseline, year 1, and year 3. **C,** Blood eosinophils in healthy controls at baseline and participants with asthma at baseline and year 3. **D,** Sputum eosinophils in healthy controls at baseline and participants with asthma at baseline, year 1, and year 3. For all figures, the horizontal dotted line indicates the cutoff classification value for high levels: high serum EPX was 17.1 ng/mL, calculated from the 95% value of the healthy controls; high blood eosinophils were 300 cells/μL; high sputum EPX was 32.3 ng/mL, calculated from the 95% value of the healthy controls; and high sputum eosinophils were 2%. Brackets and percentages identify the subset of participants classified with high eosinophil and EPX measures. *Eos*, Eosinophil.

The patients with asthma were older and heavier than the healthy control subjects (Table I). To calculate reference intervals for serum and sputum EPX, we followed the guidelines of the national committee for clinical laboratory standards.^30^ Serum EPX values in healthy subjects were log-transformed to normalize their distribution, and a value of 17.1 ng/mL was established as the upper 95th percentile value and the upper limit of normal (Fig 1, A). In a similar approach for sputum EPX, a value of 32.3 ng/mL was established as the upper 95th percentile value and the upper limit of normal (Fig 1, B). At the baseline visit for the patients with asthma, the mean EPX level in serum was 20-fold lower than in sputum (see Table E1 in this article’s Online Repository at www.jacionline.org).

To determine the prevalence of high EPX levels in serum and sputum in asthma, we calculated the percentage of participants with EPX values higher than the upper limit of normal at the baseline and yearly visits. In this way, we found that the prevalence of high serum EPX levels in asthma at baseline was 31%, which was similar to the 36% prevalence for high blood eosinophil counts (Fig 1, A and C), and the prevalence of high serum EPX was 25% and 27% at the year 1 and year 3 visits, respectively (Fig 1, A). Serum EPX was undetectable (<0.1 μg/L) in 17% of healthy controls at baseline, 8% of participants with asthma at baseline, 7% of participants with asthma at year 1, and 11% of participants with asthma at year 3. We also found that the prevalence of high sputum EPX levels in asthma at baseline was 53%, which was higher than the 32% prevalence for high sputum eosinophil counts (Fig 1, B and D), and the prevalence of high sputum EPX was 36% and 45% at the year 1 and year 3 visits, respectively (Fig 1, B and D). Sputum EPX was undetectable in 81% of healthy controls at baseline, 22% of participants with asthma at baseline, 49% of participants with asthma at year 1, and 33% of participants with asthma at year 3. In a separate analysis of patients with serum and sputum EPX measures at all time points (n = 173), we found that the prevalence of high serum EPX and high sputum EPX was similar to the prevalence values shown in Fig 1, A and B (data not shown). Taken together, these data indicate that high serum EPX values occur less frequently in asthma than high sputum EPX values and that sputum EPX levels are a more sensitive biomarker of airway eosinophilic inflammation than sputum eosinophil counts.

### Concordance between EPX and eosinophils is better in blood than in sputum

**FIG 2.**
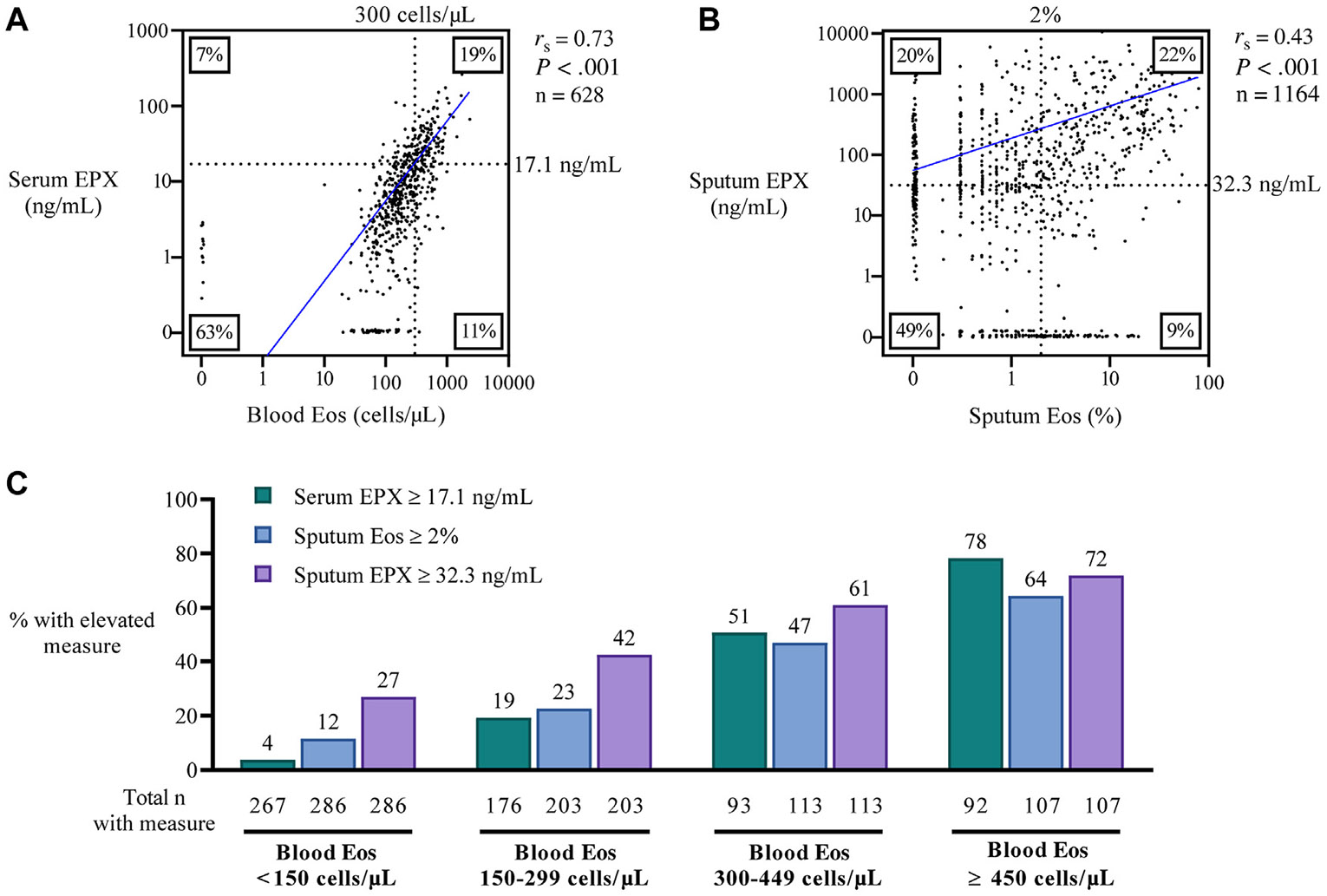
Sputum EPX identifies airway eosinophilic inflammation that can be misclassified by eosinophil counts. **A,** Correlation between blood eosinophils and serum EPX. **B,** Correlation between sputum eosinophils and sputum EPX. For Fig 2, A and B: the Spearman correlation (*r*_s_) was calculated between continuous measures and line of best fit plotted in *solid blue*. Dotted lines indicate the cutoff classification value for high/low eosinophil and EPX categorization calculated from the 95% value of the healthy controls. Boxed percentages indicate the proportion of paired samples with high/low categorization. **C,** The prevalence of high serum EPX, sputum eosinophils, and sputum EPX in patients with asthma across a range of blood eosinophil counts. The x-axis shows data for serum EPX, sputum EPX, and sputum eosinophil percentage in 4 patient subgroups classified by blood eosinophil counts that are very low (<150 cells/μL), low (between 150 and 299 cells/μL), high (counts between 150 and 299 cells/μL), and very high (>450 cells/μL). The y-axis is a 0 to 100 percentage scale of patients in each blood eosinophil subgroup who have elevated values for serum EPX, sputum EPX, or sputum eosinophils. *Eos*, Eosinophil.

To further explore relationships between eosinophil and EPX values in blood and sputum, we analyzed correlations between EPX and eosinophils in blood and sputum. In these analyses we combined all data from paired samples collected at baseline, year 1, and year 3 visits. We found that serum EPX values correlated strongly with blood eosinophil counts (Fig 2, A) and there was a high degree of concordance in high/low values for serum EPX and high/low values for blood eosinophils. Specifically, concordant high or low classification of serum EPX and blood eosinophils was seen in 518 of 628 (82%) paired samples. In contrast, the correlation between sputum EPX and sputum eosinophils was weaker, and concordance in high/low values for sputum EPX and high/low values for sputum eosinophils was lower than the same measures in blood (Fig 2, B). Specifically, concordant high or low classification of sputum EPX and sputum eosinophils was seen in 824 of 1164 (71%) paired samples.

### Blood eosinophils can misclassify airway eosinophil inflammation

Because blood eosinophils are currently used as a biomarker of airway eosinophil inflammation and type 2 inflammation, we examined how well blood eosinophil counts detect airway eosinophilic inflammation. For this analysis, we divided blood eosinophil categories into 4 subgroups (<150 cells/μL, 150-299 cells/μL, 300-449 cells/μL, and ≥450 cells/μL). For patients whose blood eosinophil counts were less than 150 cells/μL, we found that 27% had high sputum EPX values, and for those whose blood eosinophil counts were between 150 and 299 cells/μL, we found that 42% had high sputum EPX values (Fig 2, C). Thus, airway eosinophilic inflammation—as detected by sputum EPX measures—occurs frequently in patients with normal blood eosinophil counts.

### Sputum eosinophils and EPX predict outcomes of asthma severity better than blood eosinophils and EPX

Sputum measures of eosinophils and EPX predicted exacerbation-prone status (2 or more exacerbations per year) better than blood measures of eosinophils and EPX (see Table E2 in this article’s Online Repository at www.jacionline.org). Similarly, sputum measures of eosinophils and EPX predicted a low FEV_1_ (<60% predicted) better than blood measures of eosinophils and EPX (Table E2).

### Patients with persistently high sputum EPX have severe airflow obstruction, frequent exacerbations, and airway mucus plugs

**FIG 3.**
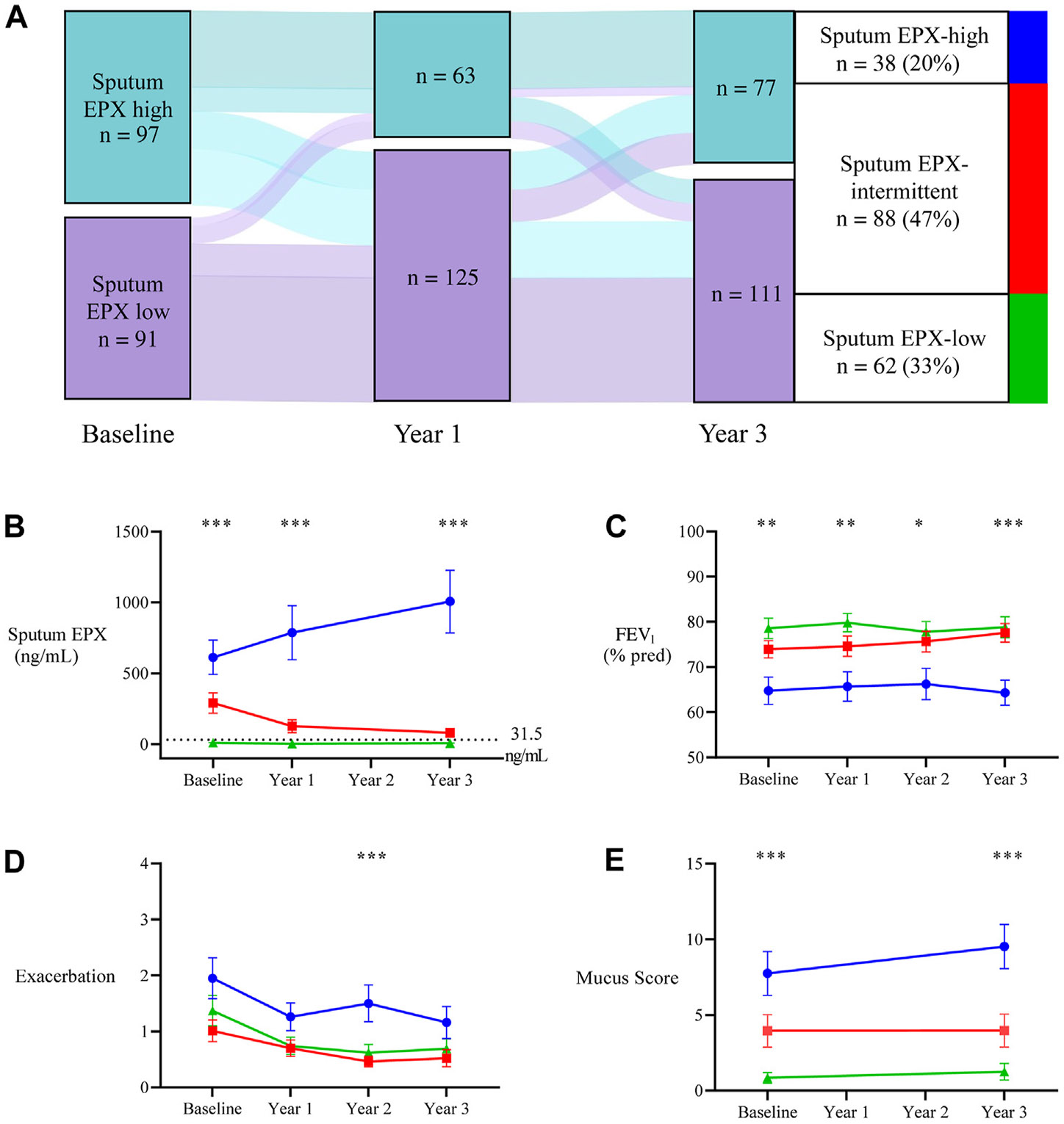
Persistently high sputum EPX is associated with airflow obstruction, frequent exacerbations, and airway mucus plugs. **A,** Sankey plot showing the change in sputum EPX in participants from baseline to year 3 and identifying subgroups with persistently high sputum EPX levels (Sputum EPX-high, *blue*), intermittently high sputum EPX levels (Sputum EPX-intermittent, *red*), and persistently low sputum EPX levels (Sputum EPX-low, *green*). **B,** Sputum EPX measures over time plotted by longitudinal sputum EPX subgroups. Horizontal dotted line indicates the high sputum EPX cutoff classification value calculated from the 95% value of the healthy controls. **C,** FEV_1_% predicted) over time plotted by longitudinal sputum EPX subgroups. **D,** Exacerbations over time plotted by longitudinal sputum EPX subgroups. **E,** Mucus plug score over time plotted by longitudinal sputum EPX subgroups. For Fig 3, B to E: mean (*dot*) and SE (*error bars*) are plotted. Comparisons between longitudinal sputum EPX subgroups were calculated using ANOVA for continuous variables with symmetric distributions and the Kruskal-Wallis test for continuous variables with skewed distributions. *P < .05; **P < .005, ***P < .0005.

To analyze sputum EPX over time, we examined data from 188 participants with asthma who had sputum EPX measures at all time points and who were not on an eosinophil-targeted biologic therapy. A Sankey plot analysis of these patients revealed that 20% had persistently high sputum EPX levels (“persistently high sputum EPX”), 47% had intermittently high sputum EPX levels (“intermittent sputum EPX”), and 33% had persistently low sputum EPX levels (“persistently low sputum EPX”) (Fig 3, A). Compared with the persistently low sputum EPX subgroup, baseline phenotyping data showed that the persistently high sputum EPX subgroup had higher values for type 2 biomarkers in blood (eosinophils and total IgE), sputum (eosinophils), and fractional exhaled nitric oxide. The persistently high sputum EPX subgroup also had clinical trait evidence of a type 2 endotype (nasal polyps) and more severe asthma, as evidenced by lower FEV_1_% predicted, more exacerbations, and higher mucus plug score compared with the persistently low sputum EPX subgroup (Table II; Fig 3). Plots of the 3-year longitudinal data showed that the mean EPX concentration in the persistently high sputum EPX group was several orders of magnitude higher than the EPX concentration in the persistently low sputum EPX group (Fig 3, B). The mean values for FEV_1_, exacerbations, and airway mucus plugging in the persistently high sputum EPX group, were also worse than in the persistently low sputum EPX group (Fig 3, C-E). In a linear mixed-effects model with a random effect for participant, we found that changes in sputum EPX were significantly associated with changes in FEV_1_ in the intermittent sputum EPX group (10 ng/mL increase in sputum EPX is associated with a 2.3% decrease in FEV_1_; *P* = .045).

### Incomplete normalization of airway eosinophilic inflammation in mepolizumab-treated patients

**FIG 4.**
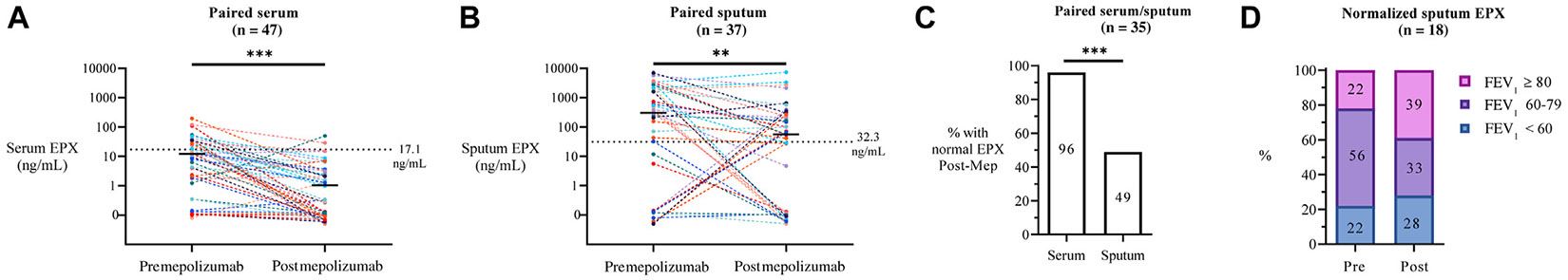
Mepolizumab normalizes serum but not sputum EPX. **A,** Serum EPX pre- and postmepolizumab. **B,** Sputum EPX pre- and postmepolizumab. For Fig 4, A and B: paired analysis of the most recent premepolizumab and the final postmepolizumab values was done with the McNemar test. Median values are plotted with solid lines. Horizontal dotted line indicates the cutoff classification value for high EPX calculated from the 95% value of the healthy controls. **C,** EPX normalization in serum and sputum. EPX normalization was defined as EPX values below the 95% threshold of healthy controls at the final postmepolizumab measure. **D,** Comparison of FEV_1_ pre- and postmepolizumab in patients with sputum EPX normalization. FEV_1_% predicted categories were compared at the most recent premepolizumab and the final postmepolizumab values in participants with sputum EPX normalization. For Fig 4, C and D: paired analysis of categorical data was done with the McNemar test. **P < .005, ***P < .0005.

To analyze the effects of mepolizumab on blood and airway measures of eosinophilic inflammation, we examined EPX data from 59 participants who had been started on mepolizumab during enrollment in SARP-3 (see Fig E4 in this article’s Online Repository at www.jacionline.org). We noticed that asthma severity was worse in these 59 patients than in the overall SARP-3 cohort (Table III). We found that serum EPX levels decreased to normal levels with mepolizumab treatment in 96% of these patients with paired pre- and postserum samples (Fig 4, A). In contrast, sputum EPX levels decreased to normal levels with mepolizumab treatment in only 49% of these patients (Fig 4, B). In addition, we found that blood eosinophil levels decreased to less than 300 cells/μL with mepolizumab treatment in 100% (54 of 54) and sputum eosinophil levels decreased to less than 2% with mepolizumab in 75% (30 of 40) of these patients. In the 18 patients whose sputum EPX normalized with mepolizumab treatment, most continued to have moderate to severe airflow obstruction (Fig 4, D). This study was not powered to make comparisons between participants whose sputum EPX did and did not normalize after mepolizumab. However, compared with patients whose sputum EPX normalized with mepolizumab treatment, those whose sputum EPX did not normalize had higher premepolizumab sputum EPX concentrations (1775 ± 2069 vs 511 ± 898 ng/mL; *P* = .023), lower postmepolizumab FEV_1_% predicted (56.0 ± 19.0 vs 74.9 ± 21.3; *P* = .007), higher postmepolizumab mucus plug scores (10.9 ± 2.9 vs 3.5 ± 4.7; *P* = .006), and fewer oral corticosteroid discontinuations (0 of 10 vs 4 of 7; *P* = .016).

## DISCUSSION

EPX, an abundant granule protein in eosinophils, is attractive as a protein biomarker of eosinophil inflammation, but its specific advantages over measures of eosinophil counts have not been fully evaluated previously. Here, we leveraged the large bio-specimen biobank of the SARP-3 cohort and the deep phenotyping of this cohort to comprehensively evaluate EPX and eosinophils as biomarkers of systemic and airway eosinophilic inflammation in asthma. We show that serum EPX is a valid protein biomarker of systemic eosinophilic inflammation, that sputum EPX levels are a more sensitive biomarker of airway eosinophilic inflammation than sputum eosinophils, and that blood eosinophils frequently misclassify airway eosinophilic inflammation. We also show that although mepolizumab usually normalizes systemic eosinophilic inflammation, it frequently fails to normalize airway eosinophilic inflammation.

Our finding that serum EPX levels correlate very well with blood eosinophils in cross-sectional analyses at multiple time points is important because it provides supporting evidence that serum EPX is a valid surrogate for measures of blood eosinophil counts in asthma. There are scenarios in which serum has been collected from participants in research studies, but blood eosinophil counts have not been measured.^31^ It is not possible to retroactively measure blood eosinophil counts, but if banked serum is available, then our data support use of serum EPX as a valid measure of systemic eosinophilic inflammation.

The correlation between sputum EPX and sputum eosinophils was relatively weak mainly because there were instances where sputum EPX levels were high when sputum eosinophil values were low. There are several reasons for such discordance. First, unlike automated blood eosinophil counts, sputum eosinophils are counted manually using cytospin slide preparations. In these slides, the eosinophils do not always retain their cell integrity after processing and can be difficult to distinguish from neutrophils, which results in interobserver variability in eosinophil counts.^11,32-35^ In addition, degranulated eosinophils may not be recognized as eosinophils in these cytospin preparation but are detected by measures of EPX.^6,36,37^ Indeed, detection of tissue eosinophil granule proteins in the absence of tissue eosinophilia is reported in many diseases, including atopic dermatitis, chronic urticaria, eosinophilic endomyocardial disease, eosinophilic gastrointestinal disease, and nasal polyposis,^34,38-41^ and EPX immunostaining is better than eosinophil counts in differentiating eosinophilic esophagitis from gastroesophageal reflux disease.^39^ Taken together, our data and the literature indicate that measures of EPX have advantages over measures of eosinophil counts in diagnosing eosinophilic inflammation in tissues, including in sputum.

In considering the limitations of our work, we note that the levels of EPX that are measured in sputum reflect protein that is present in airway secretions because of the secretion by eosinophils activated because of asthma pathobiology and because of the sputum processing procedures. Our methods do not allow an analysis of the relative contribution of these factors, but the sputum processing protocol used was originally optimized to minimize cell disruption or activation.^27^ Liquefaction of sputum in this way provides a sputum cell suspension for cytocentrifugation while also allowing for the remaining sputum to be centrifuged at relatively low speed to generate a sputum supernatant. Although it is possible that eosinophils or other cells in the sputum may be activated to some extent by this processing protocol, images of the cytocentrifuged sputum consistently show intact cells, suggesting that this method of sputum processing does not provide a strong cell activation stimulus. We acknowledge that there are instances in which cells appear disrupted and that these disrupted cells may represent cells activated by the processing protocol as well as cells that are biologically activated in transit to the lumen.

Blood eosinophil counts misclassified airway eosinophilic inflammation in a sizeable subgroup of patients.^7^ For example, we found that a third of patients with blood eosinophil counts less than 150 cells/μL had high sputum EPX values and half of those with counts between 150 and 299 cells/μL had high sputum EPX values. Thus, blood eosinophil values can be an insensitive biomarker of airway eosinophilic inflammation. This finding may explain why drugs that block ligands and receptors in the type 2 inflammation cascade are effective in blood eosinophil–low patients with asthma, albeit with smaller effect sizes than in blood eosinophil–high patients.^28,42^ Further investigation of their potential broad-based anti-inflammatory effects is needed, but it is likely that these drugs are demonstrating efficacy in patients who have airway eosinophilic inflammation not reflected by their blood eosinophil counts.

Patients with persistently high sputum EPX levels over time in SARP-3 were characterized by multiple markers of severe disease, including severe airflow obstruction and exacerbations. These data prompt consideration that EPX has a pathophysiologic role as a driver of airflow obstruction and/or exacerbations in asthma. Functionally, EPX has direct toxic effects on cells and proteins related to its peroxidation of halides.^43^ These reactions generate hypothiocyanous and hypobromous acids, leading to downstream oxidative modifications including oxidation of thiol groups, carbamylation, and formation of bromotyrosine.^23,44,45^ EPX also generates reactive oxygen species and oxidants, which leads to oxidation and nitration of tyrosine and lipids.^46,47^ These products of EPX activity are implicated in the pathogenesis of type 2 inflammation, airway remodeling, and mucus plugging in asthma.^26,43,44^ In addition, mouse models of severe asthma and EPX knockout have demonstrated roles for EPX in goblet cell metaplasia, epithelial cell mucin accumulation, and mucus production.^48,49^ Furthermore, EPX reduces protein phosphatase 2A activity via phosphorylation, resulting in reduced glucocorticoid receptor nuclear translocation and corticosteroid insensitivity.^50,51^ The plausible role of EPX as a pathologic mediator of asthma suggests that targeting EPX could be a rational approach to treating severe forms of asthma. Indeed, inhibitors of myeloperoxidase are being developed as a treatment strategy for neutrophil-associated lung disease.^52^ Relevant here, though, is our finding that normalization of sputum EPX by mepolizumab did not result in normalization of lung function. Therefore, despite its strong correlation with FEV_1_, EPX is not the only mediator of pathologic airflow obstruction in asthma.

Analysis of blood and airway measures of EPX in a subset of 59 patients with asthma, who were started on mepolizumab between the time they enrolled in SARP-3 and their year 3 visit, revealed discordance in the effects of mepolizumab on systemic and airway measures of eosinophilic inflammation. Specifically, serum EPX levels normalized in nearly all of these patients, but sputum EPX normalized in only half of them. Thus, mepolizumab is highly effective in normalizing systemic inflammation but not as effective at normalizing airway eosinophilic inflammation. Other studies have also noted this discrepancy for the effects of IL-5 inhibitors on blood and tissue eosinophilia.^53-61^ For example, in previous studies of mepolizumab, it was found that mature eosinophils are depleted in blood, but were reduced only by 79% to 94% in bronchoalveolar lavage fluid and 50% to 55% in bronchial biopsy tissue.^54,62^ Notably, mepolizumab does not suppress eosinophil progenitors^59,63,64^ or airway eosinophil activation,^65,66^ and IL-5 knockout mice can develop blood and tissue eosinophils through homeostatic IL-5–independent mechanisms, including IL-5 coredundancy with IL-3 and GM-CSF and loss of IL-5Rα in tissue-resident eosinophils.^8^ Transcriptomic analysis has also identified mechanisms of eosinophil activation, epithelial inflammation, and mucus production that are not suppressed by mepolizumab.^67^ Targeting IL-5R with benralizumab suppressed serum EDN, another eosinophil granule protein, more slowly and incompletely than blood eosinophils, although EDN is also expressed at lower levels by neutrophils and by liver cells.^68,69^ Thus, although it is possible that higher doses of mepolizumab or inhibition of the IL-5R might more effectively suppress airway eosinophilic inflammation,^59,70,71^ it is also plausible that IL-5–independent mechanisms contribute to persistent airway eosinophilic inflammation in patients treated with IL-5 inhibitors.

In summary, EPX is a valid protein biomarker of systemic eosinophilic inflammation in asthma, and EPX levels in sputum are more sensitive than eosinophil measures in sputum as a biomarker of airway eosinophilic inflammation. Airway eosinophilic inflammation, as measured by EPX measures, reveals the insensitivity of blood eosinophils as a biomarker of airway eosinophilia and shows that mepolizumab does not always normalize airway eosinophilic inflammation.

## Supplementary Material

1

## Figures and Tables

**TABLE I.** Clinical characteristics of healthy participants and participants with asthma

Characteristics	Healthy—UCSF (n = 75)	Healthy—SARP (n = 35)	Asthma—SARP (n = 479)
Age (y), mean ± SD	40.6 ± 14.7	40.1 ± 12.9	47.4 ± 13.8
Sex, female, n (%)	40 (52.6)	18 (51.4)	314 (65.6)
BMI, mean ± SD	25.27 ± 5.56	27.11 ± 5.05	32.50 ± 8.32
Race, n (%)			
White	54 (71.0)	21 (60.0)	309 (64.5)
Black	4 (5.3)	6 (17.1)	114 (23.8)
Asian	13 (17.1)	3 (8.6)	18 (3.8)
Other/multiple	5 (6.6)	5 (14.3)	38 (7.9)
Hispanic, n (%)	7 (9.2)	0 (0.0)	16 (3.3)
Pre-BD FEV_1_ (% predicted), mean ± SD	98.43 ± 11.52	98.43 ± 11.66	72.15 ± 19.62
Pre-BD FVC (% predicted), mean ± SD	100.51 ± 12.52	99.89 ± 13.69	84.61 ± 17.29

Data are reported as mean ± SD for continuous variables and n (%) for categorical variables.

*BD*, Bronchodilator; *BMI*, body mass index; *FVC*, forced vital capacity.

**TABLE II.** Clinical characteristics of longitudinal sputum EPX subgroups

Characteristics	Sputum EPX-high (n = 38)	Sputum EPX-intermittent (n = 88)	Sputum EPX-low (n = 62)	*P* value*
*Demographic characteristics*				
Age (y), mean ± SD	51.41 ± 13.44	48.13 ± 14.76	46.47 ± 13.26	.233
Sex, female, n (%)	23 (60.5)	58 (65.9)	43 (69.4)	.664
BMI, mean ± SD	30.97 ± 7.24	31.90 ± 8.40	32.59 ± 8.12	.623
Race, n (%)				.239
White	27 (71.1)	56 (63.6)	46 (74.2)	
Black	5 (13.2)	21 (23.9)	12 (19.4)	
Asian	4 (10.5)	3 (3.4)	1 (1.6)	
Other	2 (5.3)	8 (9.1)	3 (4.8)	
Hispanic, n (%)	0 (0.0)	2 (2.3)	1 (1.6)	.646
*Type 2 inflammation features*				
Blood eosinophils (cells/μL), median (quartiles)				
Baseline	371.0 (264.4-565.7)	227.6 (148.0-370.9)	156.0 (90.5-243.5)	<.001
Year 3	343.5 (245.5-515.9)	198.0 (134.0-329.6)	134.7 (78.8-250.6)	<.001
Sputum eosinophils (%), median (quartiles)				
Baseline	2.1 (0.5-13.7)	1.7 (0.4-4.7)	0.3 (0.0-0.6)	<.001
Year 1	5.7 (1.3-15.6)	1.2 (0.3-3.7)	0.3 (0.0-0.8)	<.001
Year 3	2.2 (0.3-18.1)	0.9 (0.2-3.5)	0.2 (0.0-0.6)	<.001
Feno (ppb), median (quartiles)				
Baseline	25.5 (15.8-47.5)	23.5 (14.0-39.3)	18.0 (11.0-23.0)	.004
Year 1	29.5 (17.8-67.0)	23.0 (15.5-40.5)	17.0 (12.0-24.0)	<.001
Total IgE, median (quartiles)	193.1 (57.8-566.0)	180.3 (88.9-392.9)	81.2 (27.6-206.5)	.003
Nasal polyps, n (%)	13 (34.2)	18 (20.5)	8 (12.9)	.001

Data are reported as mean ± SD for continuous variables with symmetric distribution, median (quartiles) for continuous variables with skewed distribution, and n (%) for categorical variables.

*BMI*, Body mass index; *Feno*, fractional exhaled nitric oxide; *ppb*, parts per billion.

* *P* values were calculated using ANOVA for continuous variables with symmetric distribution, the Kruskal-Wallis test for continuous variables with skewed distribution, and the χ^2^ test for categorical variables.

**TABLE III.** Characteristics of participants treated with mepolizumab

Characteristics	SARP-3 asthma (n = 427)	Mepolizumab (n = 59)	*P* value
*Demographic characteristics*			
Age (y), mean ± SD	46.8 ± 14.0	51.6 ± 15.5	.014
Sex, female, n (%)	276 (65)	42 (70)	.196
BMI, mean ± SD	32.6 ± 8.5	31.3 ± 7.8	.260
Race, n (%)			.034
White	265 (62)	47 (78)	
Black	111 (26)	7 (12)	
Asian	15 (4)	2 (3)	
Other/multiple	33 (8)	4 (7)	
Hispanic, n (%)	15 (4)	2 (3)	1.000
*Asthma characteristics*			
Daily OCS, n (%)	51 (12)	27 (47)	<.001
ACT*, median (quartiles)	18.0 (14.0-21.0)	14.0 (9.5-18.0)	<.001
Pre-BD FEV_1_ (% predicted), mean ± SD	73.3 ± 19.27	61.29 ± 21.27	<.001
Annualized exacerbation rate, median (quartiles)	1 (0-2)	2 (0-4)	.209

Data from most recent visit before starting mepolizumab. Data are reported as mean ± SD for continuous variables with symmetric distribution, median (quartiles) for continuous variables with skewed distribution, and n (%) for categorical variables.

*ACT*, Asthma control test; *BD*, bronchodilator; *BMI*, body mass index; *OCS*, oral corticosteroid.

* Scores on ACT range from 25 to 5, with lower scores indicating worse asthma control.

## Clinical implications:

EPX usefully complements eosinophil counts as a biomarker of eosinophilic inflammation in asthma. Blood-based measures of eosinophilic inflammation in patients with asthma can misclassify airway eosinophil endotypes.

## Abbreviations used

EDN: Eosinophil-derived neurotoxin
EPX: Eosinophil peroxidase
SARP: Severe Asthma Research Program
UCSF: University of California San Francisco

## References

[1] Fahy JV. Type 2 inflammation in asthma—present in most, absent in many. Nat Rev Immunol 2015;15:57–65. 25534623 10.1038/nri3786 PMC4390063

[2] Brusselle GG, Koppelman GH. Biologic therapies for severe asthma. N Engl J Med 2022;386:157–71. 35020986 10.1056/NEJMra2032506

[3] Global Initiative for Asthma. 2023. GINA main report. Available at: https://ginasthma.org/2023-gina-main-report/. Accessed June 5, 2023.

[4] Chung KF, Wenzel SE, Brozek JL, Bush A, Castro M, Sterk PJ, International ERS/ATS guidelines on definition, evaluation and treatment of severe asthma. Eur Respir J 2014;43:343–73. 24337046 10.1183/09031936.00202013

[5] Mukherjee M, Nair P. Blood or sputum eosinophils to guide asthma therapy? Lancet Respir Med 2015;3:824–5. 26493937 10.1016/S2213-2600(15)00419-1

[6] Khoury P, Akuthota P, Ackerman SJ, Arron JR, Bochner BS, Collins MH, Revisiting the NIH Taskforce on the Research needs of Eosinophil-Associated Diseases (RE-TREAD). J Leukoc Biol 2018;104:69–83. 29672914 10.1002/JLB.5MR0118-028R PMC6171343

[7] Hastie AT, Moore WC, Li H, Rector BM, Ortega VE, Pascual RM, Biomarker surrogates do not accurately predict sputum eosinophil and neutrophil percentages in asthmatic subjects. J Allergy Clin Immunol 2013;132:72–80. 23706399 10.1016/j.jaci.2013.03.044 PMC3704048

[8] Mesnil C, Raulier S, Paulissen G, Xiao X, Birrell MA, Pirottin D, Lung-resident eosinophils represent a distinct regulatory eosinophil subset. J Clin Invest 2016;126:3279–95. 27548519 10.1172/JCI85664 PMC5004964

[9] Wechsler ME, Munitz A, Ackerman SJ, Drake MG, Jackson DJ, Wardlaw AJ, Eosinophils in health and disease: a state-of-the-art review. Mayo Clin Proc 2021;96:2694–707. 34538424 10.1016/j.mayocp.2021.04.025

[10] Gleich GJ. Mechanisms of eosinophil-associated inflammation. J Allergy Clin Immunol 2000;105:651–63. 10756213 10.1067/mai.2000.105712

[11] Nair P, Ochkur SI, Protheroe C, Radford K, Efthimiadis A, Lee NA, Eosinophil peroxidase in sputum represents a unique biomarker of airway eosinophilia. Allergy 2013;68:1177–84. 23931643 10.1111/all.12206 PMC3788081

[12] Nazaroff CD, LeSuer WE, Masuda MY, Pyon G, Lacy P, Jacobsen EA. Assessment of lung eosinophils in situ using immunohistological staining. Methods Mol Biol 2021;2223:237–66. 33226599 10.1007/978-1-0716-1001-5_17 PMC7869952

[13] Makiya MA, Khoury P, Kuang FL, Mata AD, Mahmood S, Bowman A, Urine eosinophil-derived neurotoxin: a potential marker of activity in select eosinophilic disorders. Allergy 2023;78:258–69. 35971862 10.1111/all.15481 PMC11452843

[14] Abu-Ghazaleh RI, Dunnette SL, Loegering DA, Checkel JL, Kita H, Thomas LL, Eosinophil granule proteins in peripheral blood granulocytes. J Leukoc Biol 1992;52:611–8. 1464733 10.1002/jlb.52.6.611

[15] Bjornsson E, Janson C, Hakansson L, Enander I, Venge P, Boman G. Eosinophil peroxidase: a new serum marker of atopy and bronchial hyper-responsiveness. Respir Med 1996;90:39–46. 8857325 10.1016/s0954-6111(96)90243-7

[16] Keatings VM, Barnes PJ. Granulocyte activation markers in induced sputum: comparison between chronic obstructive pulmonary disease, asthma, and normal subjects. Am J Respir Crit Care Med 1997;155:449–53. 9032177 10.1164/ajrccm.155.2.9032177

[17] Frederick JM, Warner JO, Jessop WJ, Enander I, Warner JA. Effect of a bed covering system in children with asthma and house dust mite hypersensitivity. Eur Respir J 1997;10:361–6. 9042633 10.1183/09031936.97.10020361

[18] Sanz ML, Parra A, Prieto I, Diéguez I, Oehling AK. Serum eosinophil peroxidase (EPO) levels in asthmatic patients. Allergy 1997;52:417–22. 9188923 10.1111/j.1398-9995.1997.tb01021.x

[19] Parra A, Sanz ML, Vila L, Prieto I, Diéguez I, Oehling AK. Eosinophil soluble protein levels, eosinophil peroxidase and eosinophil cationic protein in asthmatic patients. J Investig Allergol Clin Immunol 1999;9:27–34. 10212854

[20] Krug N, Napp U, Enander I, Eklund E, Rieger CH, Schauer U. Intracellular expression and serum levels of eosinophil peroxidase (EPO) and eosinophil cationic protein in asthmatic children. Clin Exp Allergy 1999;29:1507–15. 10520079 10.1046/j.1365-2222.1999.00680.x

[21] Lönnkvist K, Halldén G, Dahlén SE, Enander I, van Hage-Hamsten M, Kumlin M, Markers of inflammation and bronchial reactivity in children with asthma, exposed to animal dander in school dust. Pediatr Allergy Immunol 1999;10:45–52. 10410917 10.1034/j.1399-3038.1999.101001.x

[22] Metso T, Rytilä P, Peterson C, Haahtela T. Granulocyte markers in induced sputum in patients with respiratory disorders and healthy persons obtained by two sputum-processing methods. Respir Med 2001;95:48–55. 11207017 10.1053/rmed.2000.0970

[23] Aldridge RE, Chan T, van Dalen CJ, Senthilmohan R, Winn M, Venge P, Eosinophil peroxidase produces hypobromous acid in the airways of stable asthmatics. Free Radic Biol Med 2002;33:847–56. 12208372 10.1016/s0891-5849(02)00976-0

[24] Rank MA, Ochkur SI, Lewis JC, Teaford HG, Wesselius LJ, Helmers RA, Nasal and pharyngeal eosinophil peroxidase levels in adults with poorly controlled asthma correlate with sputum eosinophilia. Allergy 2016;71:567–70. 26645423 10.1111/all.12817 PMC4803514

[25] Teague WG, Phillips BR, Fahy JV, Wenzel SE, Fitzpatrick AM, Moore WC, Baseline features of the Severe Asthma Research Program (SARP III) cohort: differences with age. J Allergy Clin Immunol Pract 2018;6:545–54.e4. 28866107 10.1016/j.jaip.2017.05.032 PMC5832534

[26] Dunican EM, Elicker BM, Gierada DS, Nagle SK, Schiebler ML, Newell JD, Mucus plugs in patients with asthma linked to eosinophilia and airflow obstruction. J Clin Invest 2018;128:997–1009. 29400693 10.1172/JCI95693 PMC5824874

[27] Fahy JV, Liu J, Wong H, Boushey HA. Cellular and biochemical analysis of induced sputum from asthmatic and from healthy subjects. Am Rev Respir Dis 1993;147:1126–31. 8484620 10.1164/ajrccm/147.5.1126

[28] Menzies-Gow A, Corren J, Bourdin A, Chupp G, Israel E, Wechsler ME, Te-zepelumab in adults and adolescents with severe, uncontrolled asthma. N Engl J Med 2021;384:1800–9. 33979488 10.1056/NEJMoa2034975

[29] Makiya MA, Herrick JA, Khoury P, Prussin CP, Nutman TB, Klion AD. Development of a suspension array assay in multiplex for the simultaneous measurement of serum levels of four eosinophil granule proteins. J Immunol Methods 2014;411:11–22. 24914990 10.1016/j.jim.2014.05.020 PMC4171350

[30] Clinical and Laboratory Standards Institute. Defining, establishing, and verifying reference intervals in the clinical laboratory. Approved Guideline. 3rd ed. Wayne, Pa: Clinical and Laboratory Standards Institute; 2008. CLSI document EP28-A3c.

[31] Bose S, Bime C, Henderson RJ, Blake KV, Castro M, DiMango E, Bio-markers of type 2 airway inflammation as predictors of loss of asthma control during step-down therapy for well-controlled disease: the Long-Acting Beta-Agonist Step-Down Study (LASST). J Allergy Clin Immunol Pract 2020;8:3474–81. 32693214 10.1016/j.jaip.2020.06.067 PMC8026280

[32] Ali MM, Wolfe MG, Mukherjee M, Radford K, Patel Z, White D, A sputum bioassay for airway eosinophilia using an eosinophil peroxidase aptamer. Sci Rep 2022;12:22476. 36577785 10.1038/s41598-022-26949-7 PMC9797489

[33] D’silva L, Allen CJ, Hargreave FE, Parameswaran K. Sputum neutrophilia can mask eosinophilic bronchitis during exacerbations. Can Respir J 2007;14:281–4. 17703243 10.1155/2007/190618 PMC2676394

[34] Lal D, Wright BL, Shim KP, Zarka MA, Lee JJ, Chang YH, Eosinophil peroxidase, GATA3, and T-bet as tissue biomarkers in chronic rhinosinusitis. J Allergy Clin Immunol 2019;143:2284–7.e6. 30738839 10.1016/j.jaci.2019.01.038

[35] Stucke EM, Clarridge KE, Collins MH, Henderson CJ, Martin LJ, Rothenberg ME. The value of an additional review for eosinophil quantification in esophageal biopsies. J Pediatr Gastroenterol Nutr 2015;61:65–8. 25633495 10.1097/MPG.0000000000000740 PMC4483145

[36] Persson CG, Erjefält JS. “Ultimate activation” of eosinophils in vivo: lysis and release of clusters of free eosinophil granules (Cfegs). Thorax 1997;52:569–74. 9227728 10.1136/thx.52.6.569 PMC1758581

[37] Weller PF, Spencer LA. Functions of tissue-resident eosinophils. Nat Rev Immunol 2017;17:746–60. 28891557 10.1038/nri.2017.95 PMC5783317

[38] Miyabe Y, Kobayashi Y, Fukuchi M, Saga A, Moritoki Y, Saga T, Eosinophil-mediated inflammation in the absence of eosinophilia. Asia Pac Allergy 2021;11:e30. 34386406 10.5415/apallergy.2021.11.e30 PMC8331253

[39] Protheroe C, Woodruff SA, de Petris G, Mukkada V, Ochkur SI, Janarthanan S, A novel histologic scoring system to evaluate mucosal biopsies from patients with eosinophilic esophagitis. Clin Gastroenterol Hepatol 2009;7:749–55.e11. 19345285 10.1016/j.cgh.2009.03.022 PMC2706311

[40] Filley WV, Holley KE, Kephart GM, Gleich GJ. Identification by immunofluorescence of eosinophil granule major basic protein in lung tissues of patients with bronchial asthma. Lancet 1982;2:11–6. 6177986 10.1016/s0140-6736(82)91152-7

[41] Leiferman KM, Ackerman SJ, Sampson HA, Haugen HS, Venencie PY, Gleich GJ. Dermal deposition of eosinophil-granule major basic protein in atopic dermatitis. Comparison with onchocerciasis. N Engl J Med 1985;313:282–5. 3892296 10.1056/NEJM198508013130502

[42] Bleecker ER, FitzGerald JM, Chanez P, Papi A, Weinstein SF, Barker P, Efficacy and safety of benralizumab for patients with severe asthma uncontrolled with high-dosage inhaled corticosteroids and long-acting β2-agonists (SIROCCO): a randomised, multicentre, placebo-controlled phase 3 trial. Lancet 2016;388:2115–27. 27609408 10.1016/S0140-6736(16)31324-1

[43] Mitra SN, Slungaard A, Hazen SL. Role of eosinophil peroxidase in the origins of protein oxidation in asthma. Redox Rep 2000;5:215–24. 10994876 10.1179/135100000101535771

[44] Wang Z, DiDonato JA, Buffa J, Comhair SA, Aronica MA, Dweik RA, Eosinophil peroxidase catalyzed protein carbamylation participates in asthma. J Biol Chem 2016;291:22118–35. 27587397 10.1074/jbc.M116.750034 PMC5063994

[45] Wang Z, Xu W, Comhair SAA, Fu X, Shao Z, Bearden R, Urinary total conjugated 3-bromotyrosine, asthma severity, and exacerbation risk. Am J Physiol Lung Cell Mol Physiol 2022;323:L548–57. 36126269 10.1152/ajplung.00141.2022 PMC9602918

[46] MacPherson JC, Comhair SA, Erzurum SC, Klein DF, Lipscomb MF, Kavuru MS, Eosinophils are a major source of nitric oxide-derived oxidants in severe asthma: characterization of pathways available to eosinophils for generating reactive nitrogen species. J Immunol 2001;166:5763–72. 11313420 10.4049/jimmunol.166.9.5763

[47] Abu-Soud HM, Khassawneh MY, Sohn JT, Murray P, Haxhiu MA, Hazen SL. Peroxidases inhibit nitric oxide (NO) dependent bronchodilation: development of a model describing NO-peroxidase interactions. Biochemistry 2001;40:11866–75. 11570887 10.1021/bi011206v

[48] Jacobsen EA, Ochkur SI, Doyle AD, LeSuer WE, Li W, Protheroe CA, Lung pathologies in a chronic inflammation mouse model are independent of eosinophil degranulation. Am J Respir Crit Care Med 2017;195:1321–32. 27922744 10.1164/rccm.201606-1129OC PMC5443899

[49] Lu Y, Huang Y, Li J, Huang J, Zhang L, Feng J, Eosinophil extracellular traps drive asthma progression through neuro-immune signals. Nat Cell Biol 2021;23:1060–72. 34616019 10.1038/s41556-021-00762-2

[50] Kobayashi Y, Kanda A, Yun Y, Bui DV, Suzuki K, Sawada S, Reduced local response to corticosteroids in eosinophilic chronic rhinosinusitis with asthma. Bio-molecules 2020;10:326. 10.3390/biom10020326 PMC7072408 32085629

[51] Kobayashi Y, Kanda A, Bui DV, Yun Y, Nguyen LM, Chu HH, Omalizumab restores response to corticosteroids in patients with eosinophilic chronic rhinosinusitis and severe asthma. Biomedicines 2021;9:787. 34356851 10.3390/biomedicines9070787 PMC8301363

[52] A phase IIa randomised, double blind, placebo controlled, parallel arm, multi-centre study to evaluate the efficacy and safety of mitiperstat (AZD4831), for 12-24 weeks, in patients with moderate to severe chronic obstructive pulmonary disease (COPD). Report no. study/NCT05492877. AstraZeneca, Cambridge, UK. 2023. Available at: https://clinicaltrials.gov/ct2/show/study/NCT05492877. Accessed June 6, 2023.

[53] Kips JC, O’Connor BJ, Langley SJ, Woodcock A, Kerstjens HAM, Postma DS, Effect of SCH55700, a humanized anti-human interleukin-5 antibody, in severe persistent asthma: a pilot study. Am J Respir Crit Care Med 2003;167:1655–9. 12649124 10.1164/rccm.200206-525OC

[54] Haldar P, Brightling CE, Hargadon B, Gupta S, Monteiro W, Sousa A, Mepolizumab and exacerbations of refractory eosinophilic asthma. N Engl J Med 2009;360:973–84. 19264686 10.1056/NEJMoa0808991 PMC3992367

[55] Nair P, Pizzichini MMM, Kjarsgaard M, Inman MD, Efthimiadis A, Pizzichini E, Mepolizumab for prednisone-dependent asthma with sputum eosinophilia. N Engl J Med 2009;360:985–93. 19264687 10.1056/NEJMoa0805435

[56] Castro M, Mathur S, Hargreave F, Boulet LP, Xie F, Young J, Reslizumab for poorly controlled, eosinophilic asthma: a randomized, placebo-controlled study. Am J Respir Crit Care Med 2011;184:1125–32. 21852542 10.1164/rccm.201103-0396OC

[57] Pavord ID, Korn S, Howarth P, Bleecker ER, Buhl R, Keene ON, Mepolizumab for severe eosinophilic asthma (DREAM): a multicentre, double-blind, placebo-controlled trial. Lancet 2012;380:651–9. 22901886 10.1016/S0140-6736(12)60988-X

[58] Nair P, Wenzel S, Rabe KF, Bourdin A, Lugogo NL, Kuna P, Oral glucocorticoid-sparing effect of benralizumab in severe asthma. N Engl J Med 2017;376:2448–58. 28530840 10.1056/NEJMoa1703501

[59] Mukherjee M, Aleman Paramo F, Kjarsgaard M, Salter B, Nair G, LaVigne N, Weight-adjusted intravenous reslizumab in severe asthma with inadequate response to fixed-dose subcutaneous mepolizumab. Am J Respir Crit Care Med 2018;197:38–46. 28915080 10.1164/rccm.201707-1323OC

[60] Ojanguren I, Chaboillez S, Lemiere C. Low blood eosinophil counts are not always a reliable marker of clinical response to mepolizumab in severe asthma. J Allergy Clin Immunol Pract 2018;6:2151–3. 29715561 10.1016/j.jaip.2018.04.014

[61] Bagnasco D, Massolo A, Bonavia M, Brussino L, Bucca C, Caminati M, The importance of being not significant: blood eosinophils and clinical responses do not correlate in severe asthma patients treated with mepolizumab in real life. Allergy 2020;75:1460–3. 31773742 10.1111/all.14135

[62] Flood-Page PT, Menzies-Gow AN, Kay AB, Robinson DS. Eosinophil’s role remains uncertain as anti-interleukin-5 only partially depletes numbers in asthmatic airway. Am J Respir Crit Care Med 2003;167:199–204. 12406833 10.1164/rccm.200208-789OC

[63] Menzies-Gow A, Flood-Page P, Sehmi R, Burman J, Hamid Q, Robinson DS, Anti-IL-5 (mepolizumab) therapy induces bone marrow eosinophil maturational arrest and decreases eosinophil progenitors in the bronchial mucosa of atopic asthmatics. J Allergy Clin Immunol 2003;111:714–9. 12704348 10.1067/mai.2003.1382

[64] Sehmi R, Smith SG, Kjarsgaard M, Radford K, Boulet LP, Lemiere C, Role of local eosinophilopoietic processes in the development of airway eosinophilia in prednisone-dependent severe asthma. Clin Exp Allergy 2016;46:793–802. 26685004 10.1111/cea.12695

[65] Johansson MW, Gunderson KA, Kelly EAB, Denlinger LC, Jarjour NN, Mosher DF. Anti-IL-5 attenuates activation and surface density of β2-integrins on circulating eosinophils after segmental antigen challenge. Clin Exp Allergy 2013;43:292–303. 23414537 10.1111/j.1365-2222.2012.04065.x PMC3579563

[66] Kelly EA, Esnault S, Liu LY, Evans MD, Johansson MW, Mathur S, Mepolizumab attenuates airway eosinophil numbers, but not their functional phenotype, in asthma. Am J Respir Crit Care Med 2017;196:1385–95. 28862877 10.1164/rccm.201611-2234OC PMC5736971

[67] Jackson DJ, Bacharier LB, Gergen PJ, Gagalis L, Calatroni A, Wellford S, Mepolizumab for urban children with exacerbation-prone eosinophilic asthma in the USA (MUPPITS-2): a randomised, double-blind, placebo-controlled, parallel-group trial. Lancet 2022;400:502–11. 35964610 10.1016/S0140-6736(22)01198-9 PMC9623810

[68] Chan R, RuiWen Kuo C, Jabbal S, Lipworth BJ. Eosinophil depletion with benralizumab is associated with attenuated mannitol airway hyperresponsiveness in severe uncontrolled eosinophilic asthma. J Allergy Clin Immunol 2023;151:700–5.e10. 36400178 10.1016/j.jaci.2022.10.028

[69] Sur S, Glitz DG, Kita H, Kujawa SM, Peterson EA, Weiler DA, Localization of eosinophil-derived neurotoxin and eosinophil cationic protein in neutrophilic leukocytes. J Leukoc Biol 1998;63:715–22. 9620664 10.1002/jlb.63.6.715

[70] Laviolette M, Gossage DL, Gauvreau G, Leigh R, Olivenstein R, Katial R, Effects of benralizumab on airway eosinophils in asthmatic patients with sputum eosinophilia. J Allergy Clin Immunol 2013;132:1086–96.e5. 23866823 10.1016/j.jaci.2013.05.020 PMC4172321

[71] Sehmi R, Lim HF, Mukherjee M, Huang C, Radford K, Newbold P, Benra-lizumab attenuates airway eosinophilia in prednisone-dependent asthma. J Allergy Clin Immunol 2018;141:1529–32.e8. 29382593 10.1016/j.jaci.2018.01.008

