# Supplementary material for: Utility of eosinophil peroxidase as a biomarker of eosinophilic inflammation in asthma
Source: J Allergy Clin Immunol. Author manuscript; Available in PMC 2026 Jan 4. (PMC12765372; doi:10.1016/j.jaci.2024.03.023)
Supplement: 1 [file NIHMS2128153-supplement-1.pdf]

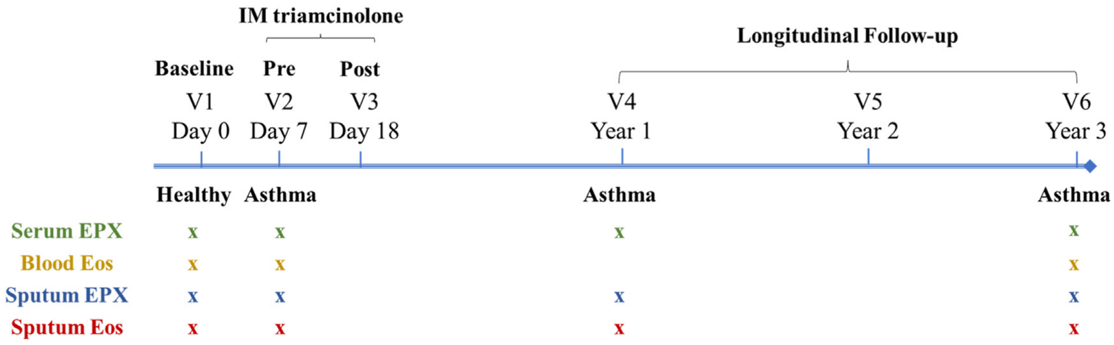

FIG E1. Schematic of the SARP-3 protocol. Analysis of serum and sputum for EPX and eosinophils was done at baseline, year 1, and year 3. Blood eosinophils were measured at baseline and year 3. Healthy controls had only baseline measures. *Eos*, Eosinophil; *IM,* intramuscular.

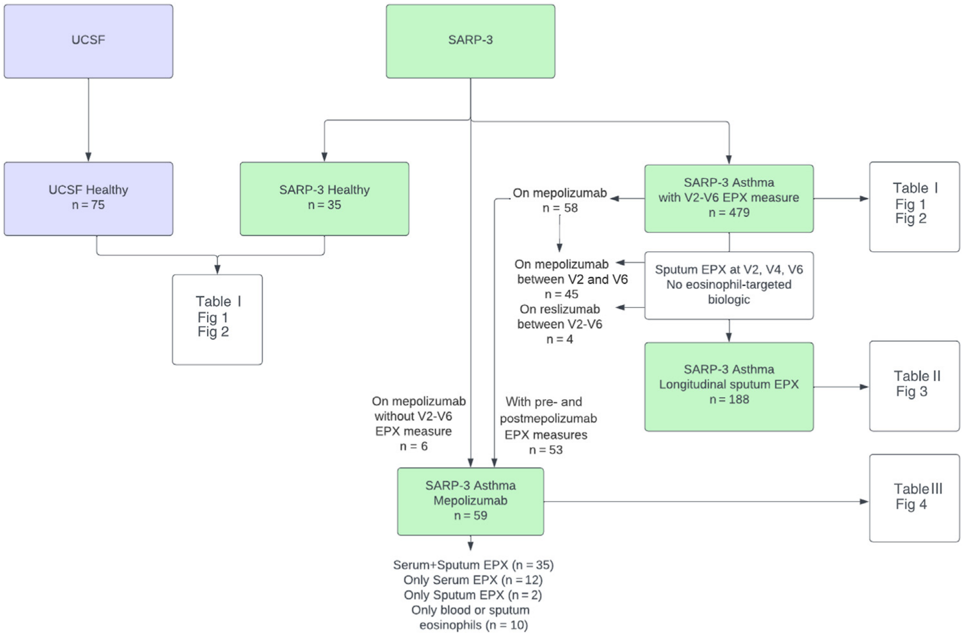

FIG E2. Participant flowchart. The flowchart illustrates the participants and samples used in different analyses.

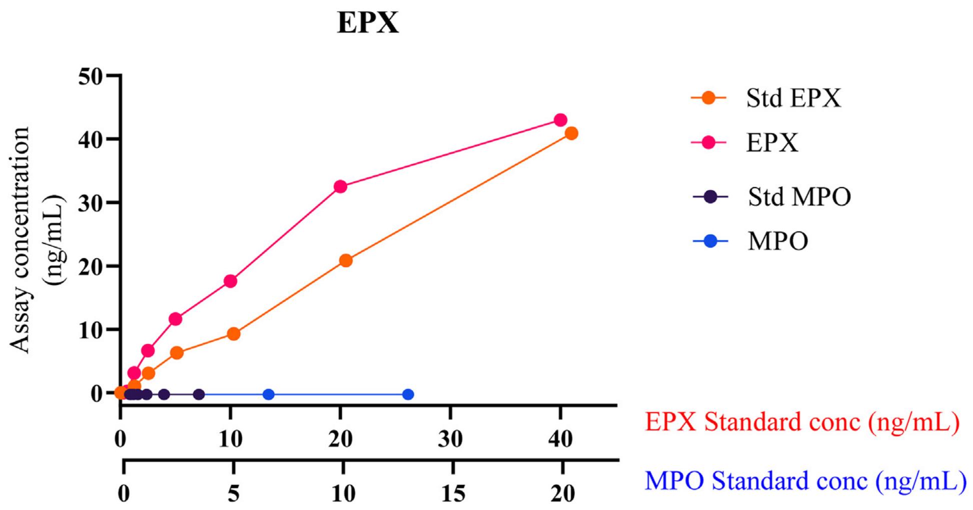

FIG E3. EPX ELISA validation assay. The EPX ELISA assay detected EPX concentration with the kit standard EPX (Std EPX, Diagnostics Development, Uppsala, Sweden) and a human recombinant EPX spike-in (EPX, Lee BioSolutions, Maryland Heights, Mo), but not kit standard myeloperoxidase (Std MPO, R&D Systems, Minneapolis, Minn) or human recombinant MPO spike-in (MPO, Abcam, Cambridge, United Kingdom). The y-axis plots the assay concentration measured with the EPX ELISA. The x-axis plots the standard concentration as determined from the kit standard or the amount spiked in.

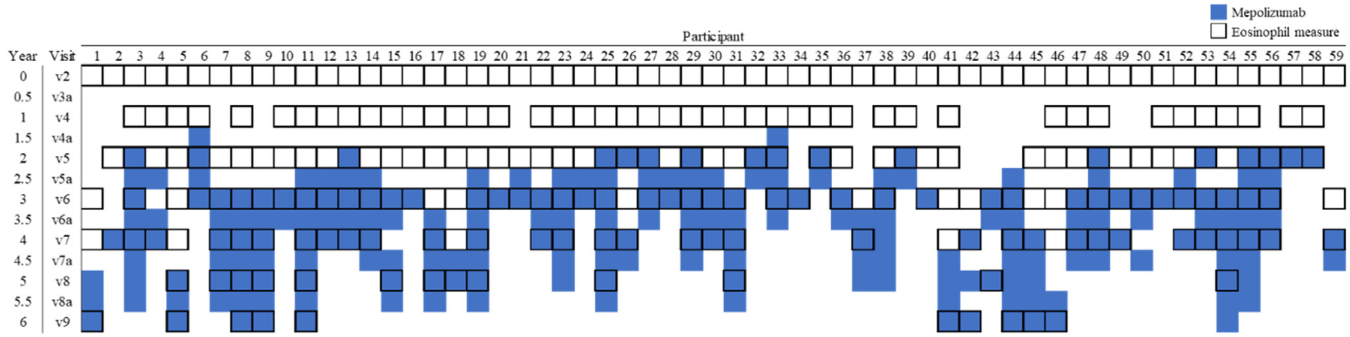

FIG E4. Schematic of mepolizumab therapy and eosinophil measures. EPX measures were analyzed in SARP-3 participants treated with mepolizumab with available pre- and postserum or sputum samples. For each participant, visits with documented mepolizumab use are filled in *blue* and eosinophil or EPX measure boxed with *solid line*.

TABLE E1. Mean and SE for eosinophil counts and EPX in the SARP-3 cohort over 3 y

Measure	Healthy	Baseline	Year 1	Year 3
Serum EPX (ng/mL)	5.1 (0.7)	17.4 (1.4)	15.7 (1.7)	14.0 (1.4)
Sputum EPX (ng/mL)	10.9 (5.7)	351.6 (45.8)	275.3 (37.2)	272.0 (47.2)
Blood eosinophil (cells/μL)	130.5 (9.6)	288.4 (12.2)		230.2 (10.8)
Sputum eosinophil (%)	0.5 (0.1)	4.3 (0.5)	4.8 (0.6)	3.8 (0.5)

TABLE E2. Comparison of blood and sputum eosinophil measures with clinical outcomes

	Exacerbations			FEV_1 (% predicted)		
**Measure**	r_s	*P*	**AUC for ≥2**	r_s	*P*	**AUC for <60%**
Blood eosinophil (cells/μL)	0.041	.271	0.531	−0.197	<.001	0.563
Serum EPX (ng/mL)	0.042	.265	0.534	−0.164	<.001	0.559
Sputum eosinophil (%)	0.107	<.001	0.571	−0.259	<.001	0.642
Sputum EPX (ng/mL)	0.162	<.001	0.599	−0.260	<.001	0.642

Correlation (r_s and *P*) between eosinophil measures and clinical outcomes was calculated using the Spearman correlation. AUC was calculated from receiver-operating curves. *AUC*, Area under the receiver-operating characteristic curve.
